# Multiscale Analysis of Reinforced Concrete Frames with Embedded Metamaterials Under Progressive Collapse

**DOI:** 10.3390/ma19112363

**Published:** 2026-06-02

**Authors:** Xu Long, Christopher Samuneti, Percy M. Iyela, Khaja Wahaajuddin Kawkabi, Prince Manyanya Ngangura, Kunjie Fan

**Affiliations:** School of Mechanics and Transportation Engineering, Northwestern Polytechnical University, Xi’an 710129, China; samuneti@mail.nwpu.edu.cn (C.S.); percyiyela@mail.nwpu.edu.cn (P.M.I.);

**Keywords:** progressive collapse, embedded metamaterials, auxetic concrete, negative Poisson’s ratio, sub-modelling, mesoscale modelling, reinforced concrete

## Abstract

Progressive collapse represents a catastrophic failure mode for reinforced concrete (RC) structures, yet the use of architected materials to mitigate this risk remains largely unexplored. This study presents a numerical feasibility investigation of RC beam–column sub-assemblages with auxetic metamaterial inserts embedded in critical joint regions. A hierarchical multiscale framework is developed to link the effective behavior of auxetic metamaterials with structure-scale collapse response. The framework couples macroscale structural analysis with mesoscale fracture simulations through a hybrid voxel–Voronoi discretization strategy. Baseline finite element models are validated against published experimental results for conventional RC specimens, while the auxetic-enhanced configurations are evaluated numerically. Under high tensile strain, the auxetic insert expands laterally because of its negative Poisson’s ratio and generates a localized confining stress field within the surrounding concrete. The simulations suggest that this mechanism may promote crack bifurcation, redistribute localized cracking into a more distributed damage pattern, and delay compressive crushing and crack coalescence. Compared with the corresponding conventional RC configurations, the auxetic-enhanced models predict a 25% increase in load redistribution capacity and a 20% enhancement in deformation ductility. These predicted improvements require future experimental validation using physical auxetic-enhanced RC specimens. The findings provide a computational basis for exploring material-by-design strategies aimed at improving the robustness of critical RC joint regions under progressive collapse demands.

## 1. Introduction

Progressive collapse, characterized by a disproportionate failure of a structural system triggered by localized damage, remains one of the most critical challenges in structural safety and resilience [1]. Traditional design philosophies, primarily the tie-force and alternative-path methods, enhance global redundancy but often fail to address the inherently brittle behavior of reinforced concrete (RC) joints under extreme load redistribution. During a column-removal scenario, beam–column joints undergo a complex transition from flexural action to catenary action (CA). The structural robustness of a frame depends strongly on the rotational capacity of these critical regions. However, standard RC details often lack the necessary ductility to accommodate these large displacements, leading to premature fracture at the critical locations of the frame.

Recent advancements in material-by-design strategies have introduced architected metamaterials as a potential solution for extreme load mitigation [2,3]. Among these materials, auxetic metamaterials, characterized by a negative Poisson’s ratio, offer unique mechanical advantages, including enhanced indentation resistance and fracture toughness. Unlike conventional materials that contract laterally under tension, auxetic inclusions expand and create a localized active confinement effect. Although the theoretical benefits of auxetic metamaterials are well-documented at the material scale, their integration into large-scale civil infrastructure remains largely unexplored.

Existing numerical approaches to structural failure generally fall into two categories. Macroscale models are computationally efficient for full-frame analysis but rely on empirical damage laws of materials that cannot capture the heterogeneous cracking physics of the joint [4,5,6,7]. In contrast, mesoscale models explicitly represent aggregates, mortar, and the interfacial transition zone, thereby providing higher physical fidelity [5]. High-fidelity numerical simulations have been shown to be essential for capturing concrete fracture behaviour [8], as macroscale models oversimplify these interactions. Furthermore, the shape and distribution of aggregates significantly influence internal stress bridges and fracture pathways. However, the computational cost of such high-fidelity models typically limits their application to small-scale specimens rather than full structural assemblies.

Recent studies have examined auxetic materials in cementitious composites and engineering applications. These studies show that auxetic components can expand laterally, improve bonding, control cracking, and absorb more energy when embedded in cementitious matrices [9,10]. However, most applications remain limited to material- or small-component-scale demonstrations. This limitation becomes important in progressive collapse, where RC joints must not only absorb energy but also sustain large rotations, redistribute load, and delay localized fracture.

Progressive collapse studies have also shown that RC frames resist column loss through a transition from flexural action to compressive arch action (CA) and tensile catenary action. These resistance mechanisms depend on reinforcement detailing, joint rotation capacity, boundary conditions, and the deformation capacity of critical joint regions [11,12]. Therefore, improving the local damage tolerance of beam–column joints remain essential for enhancing structural robustness. Mesoscale concrete studies further show that aggregates, mortar, and the interfacial transition zone (ITZ) strongly affect crack initiation, crack deflection, and fracture localization. The ITZ often acts as a weak path for damage initiation and propagation, while aggregate size, shape, and volume fraction influence stress bridging and fracture paths [13,14]. These findings support the need for a multiscale framework that links local heterogeneous fracture processes with global collapse response. However, few studies have integrated auxetic metamaterial inserts into critical RC joint regions or explained their damage-mitigation mechanism from a mesoscale perspective. This gap motivates the present numerical feasibility study. In this study, published experimental results on conventional RC sub-assemblages are used to validate the baseline finite element models. Auxetic metamaterial inserts are then introduced numerically to investigate their predicted damage-mitigation mechanism. No physical auxetic-enhanced RC specimens are tested in this work; therefore, the predicted performance improvements require future experimental confirmation.

The novelty of this work lies in an integrated numerical approach that introduces mechanically triggered auxetic inserts intended to activate under the high-strain demands of a collapse event. This mechanism is examined through a computational framework that combines hybrid voxel–Voronoi discretization with sub-modelling. The proposed method builds on the fracture modeling approach developed by Guo and Lu [15] and allows the predicted influence of auxetic inserts on crack evolution and damage distribution within RC joints to be examined. Specifically, the framework evaluates how aggregate volume fraction (AVF) and auxetic expansion may interact to influence damage localization in the joint region.

By numerically examining the relationship between metamaterial properties and structural response, this study explores a material-by-design strategy for improving critical RC joint regions under progressive collapse demands. The following sections describe the hierarchical multiscale framework, the validation of the baseline numerical models against experimental benchmarks, and the predicted influence of auxetic inserts on damage evolution and failure-mode transition.

## 2. Implementation of Macroscale and Mesoscale FE Models

### 2.1. Multiscale Numerical Framework

**Figure 1:**
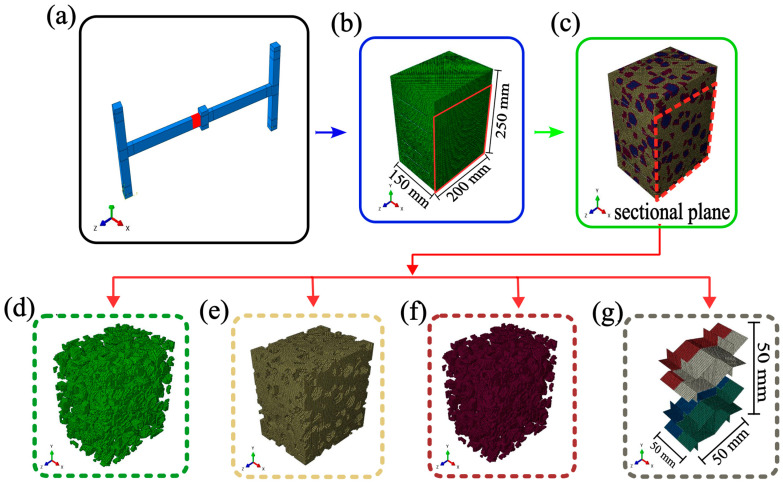
Generation of meso-structure of concrete: (**a**) macroscale model, (**b**) sub-model, (**c**) mesoscale model, (**d**) aggregate, (**e**) ITZ, (**f**) mortar, and (**g**) embedded auxetic metamaterial insert.

This study implements a hierarchical finite element framework spanning multiple scales to bridge material architecture with structural collapse response, as illustrated in Figure 1. At the macroscale, complete 3D RC beam–column frames are modeled to capture global force redistribution and the progression of collapse mechanisms following column loss. Progressing to a finer resolution, sub-models of critical beam–column joint regions are extracted from the macroscale simulations. These are driven by kinematically consistent boundary conditions, enabling high-resolution analysis of localized stress concentrations without the computational burden of a full-frame continuum model.

At the most detailed level, mesoscale models explicitly resolve the concrete’s heterogeneous microstructure, including randomly distributed aggregates, the mortar matrix, and the ITZ, alongside the embedded auxetic metamaterial inserts. Quasi-static conditions are assumed, with strain rate effects in the constitutive behaviour of materials neglected [16]. This integrated, multi-tiered approach enables a direct investigation of how micromechanical interactions, particularly the lateral expansion of the auxetic phase, translate into enhanced global structural performance and delayed failure. Each modeling tier is implemented in Abaqus/Explicit, with a one-way coupling strategy ensuring that the non-linear damage evolution at the mesoscale is physically grounded in the structural-scale loading history. Rigorous validation against experimental benchmarks for both the RC frames and the mesoscale fracture behavior ensures the computational reliability of the framework.

### 2.2. Mesoscale FE Model

The mesoscale finite element model was established with one primary objective: to resolve the fundamental mechanisms through which embedded auxetic metamaterials govern fracture processes and enhance progressive collapse resistance. The model explicitly represents the auxetic metamaterial as a distinct, active phase within the concrete matrix, enabling direct investigation of its micromechanical interactions with aggregates, mortar, and the interfacial transition zone. This explicit representation is essential for capturing the novel stress-mitigation and crack-control mechanisms introduced by the metamaterial, which are fundamentally inaccessible in homogenized continuum-based models [5,6,17,18]. The model thus provides the critical micromechanical foundation that explains the superior structural performance, enhanced ductility, stable damage progression, and increased load redistribution observed and validated at the macroscale.

A hybrid voxel-Voronoi discretization framework was implemented in Abaqus/Explicit to construct the mesoscale model, a methodology specifically designed to resolve the critical interactions between the auxetic metamaterial insert and the surrounding concrete microstructure. The procedure begins with a representative cubic domain of dimensions *L_x_ × L_y_ × L_z_*, which is discretized into a uniform grid of voxel elements. The voxel size, calibrated to between 1/4 and 1/8 of the minimum aggregate diameter [19], was critically selected to ensure the geometric fidelity required to explicitly resolve the auxetic insert’s architecture and its interface with the concrete matrix, which is the primary zone for the metamaterial’s confining and crack-bridging actions. This voxel-sized domain is then partitioned into distinct sub-regions *V_i_* (0 < *i* ≤ *N_s_*) via a Voronoi tessellation algorithm driven by randomly distributed seeds *S_i_* (0 < *i* ≤ *N_s_*). This algorithm replicates the stochastic heterogeneity of concrete [20], generating realistic aggregate, mortar, and ITZ phases. The explicit geometrical representation of these conventional phases serves one primary function: to establish a physically accurate micromechanical environment in which to isolate and quantify the active, damage-governing role of the embedded auxetic metamaterial.

The embedded auxetic metamaterial insert (Figure 1g) is the core innovative component of this framework, designed to actively govern damage progression through its negative Poisson’s ratio behaviour. The insert is assigned the effective macroscopic properties derived in Section 2.3, namely a Young’s modulus of 0.35 GPa and a Poisson’s ratio of −0.25. These properties are calibrated to represent the structural-scale expansion mechanism of its re-entrant honeycomb architecture while ensuring effective stress transfer to the surrounding concrete. The insert geometry consists of a 50 mm × 50 mm × 150 mm prism embedded within a representative volume of 150 mm × 150 mm × 150 mm and positioned adjacent to the column face to ensure engagement within the peak tensile stress zone. This placement guarantees activation during critical structural phases of plastic hinge formation and catenary action development. Under the resulting tensile strain, the auxetic mechanism drives lateral expansion, generating a localized confining stress field that directly mitigates crack propagation and enhances ductility. Collectively, the insert’s properties, geometry, and placement are selected to provide an effective mechanical interface, offering a promising strategy for metamaterial-based enhancement of structural joints against progressive collapse.

A realistic description of concrete heterogeneity requires accurate representation of both graded aggregates and the surrounding ITZs. In this study, aggregates were generated synthetically using a Fuller-type distribution curve [21], which has been widely adopted to approximate the gradation of coarse particles in normal-strength and high-strength concretes. The cumulative volume fraction function for aggregate size can be expressed as:

(1) $$P(d)\;=\;100{\left(\frac{d}{d_{max}}\right)}^{n}$$

where *P*(*d*) is the cumulative volume fraction of the aggregates with particle sizes no more than *d*, *d*_max_ is the maximum particle size of aggregates, and *n* is the empirical exponent ranging from 0.45 to 0.70. In this paper, *n* is assumed to be 0.5. The volume of graded aggregates ranging in particle size from *d_i_* to *d_i_*_+1_ is expressed as

(2) $$V_{agg}\left[d_{i},\;d_{i+1}\right]=\frac{P(d_{i+1})-P(d_{i})}{P(d_{max})-P(d_{min})}\times P_{agg}\times V_{con}=\frac{d_{i+1}^{n}-d_{i}^{n}}{d_{max}^{n}-d_{min}^{n}}\times P_{agg}\times V_{con}$$

where *d*_max_ and *d*_min_ are the maximum and minimum sizes of random aggregates, *P*_agg_ is the aggregates volume fraction, and *V*_con_ is the volume of the overall concrete. The classical size in the sieve analysis of coarse aggregates is selected considering four sieve sizes in the range of 4.75 (*d*_min_) to 19.0 (*d*_max_) [22,23]. The aggregates with sizes smaller than 4.75 mm are ignored as non-aggregate phases to ensure computational performance. The size distribution of graded aggregates in this study is presented in Table 1.

**Figure 2:**
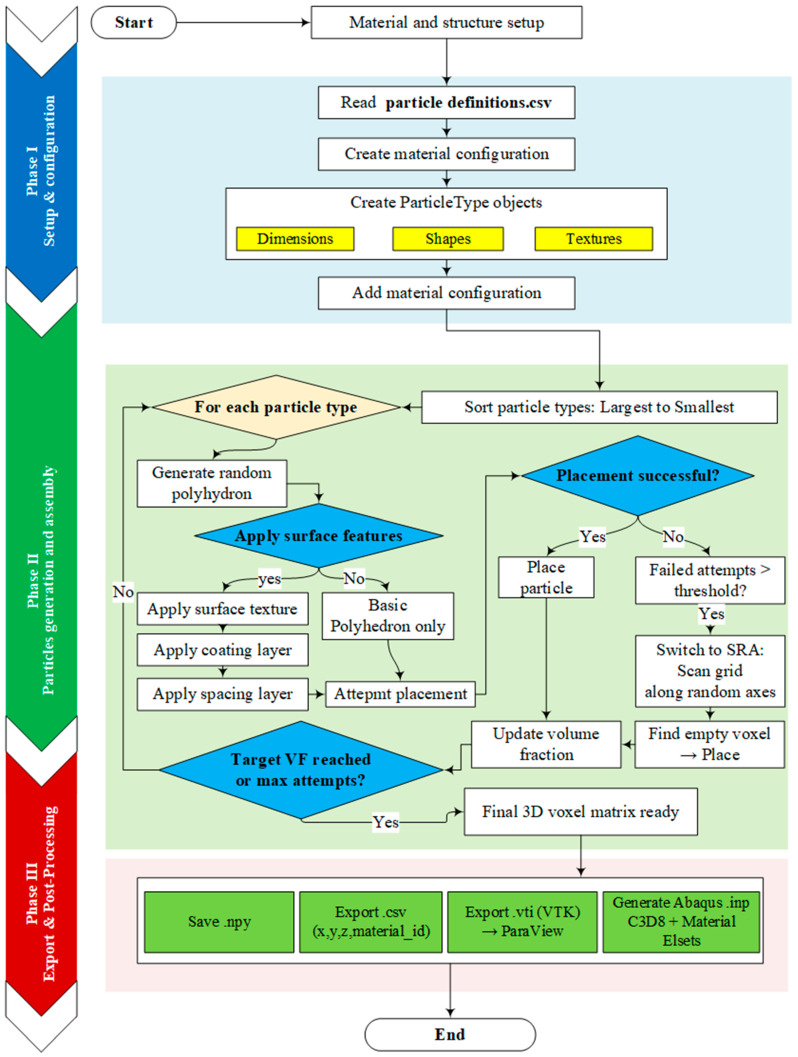
Computational workflow for mesoscale concrete generation: The flowchart illustrates the sequential algorithm for synthetic aggregate generation, placement, and discretization into a finite element mesh, utilizing a hybrid voxel-Voronoi approach.

This study develops a mesoscale framework to investigate and quantify the damage-mitigation effect of the embedded auxetic metamaterial. To achieve this, the interfacial transition zone (ITZ) is explicitly represented as a thin cohesive layer surrounding aggregates via a voxel-distance algorithm [23,24,25,26,27,28]. This controlled micromechanical environment captures the locations where crack initiation typically occurs in conventional concrete, thereby providing a reference for assessing the effect of the embedded metamaterial. Discretization of the heterogeneous system is accomplished using a uniform hexahedral background grid [29], which provides sufficient resolution to capture steep strain gradients and localized confining stress fields associated with the auxetic metamaterial’s lateral expansion under tension. Figure 2 summarizes the computational workflow used to generate the mesoscale concrete model. The procedure begins with the material and structure setup, where the particle definitions, including aggregate size, shape, and surface features, are imported from the input file and converted into particle-type objects. The particle types are then sorted from the largest to the smallest size to improve packing efficiency during aggregate placement. In the particle-generation stage, random polyhedral aggregates are generated and assigned surface features, coating layers, and spacing layers when required. Each particle is then placed into the voxel domain. If the placement fails after a prescribed number of attempts, a scanning-based random-axis search is used to identify an available voxel position. This process continues until the target aggregate volume fraction is reached or the maximum number of placement attempts is exceeded. In the final stage, the completed three-dimensional voxel matrix is exported for post-processing and finite element analysis, including material identification files, VTK files for visualization, and Abaqus input files containing C3D8 elements and material sets. This workflow ensures that the mesoscale model preserves the prescribed aggregate gradation, spatial distribution, and phase assignment required for the subsequent fracture simulations. As illustrated in Figure 2, the integrated generation process provides a computational platform for examining how the embedded metamaterial redistributes tensile stress near the ITZ and influences interfacial damage initiation. This modeling strategy enables quantitative assessment of the mechanistic link between metamaterial-induced stress redistribution and delayed crack initiation. It is noted that the auxetic insert itself is modeled as a homogenized continuum using the effective properties derived in Section 2.4.4, not as a resolved lattice geometry. The surrounding concrete microstructure (aggregates, mortar, ITZ) is explicitly resolved.

### 2.3. Macroscale FE Model

**Figure 3:**
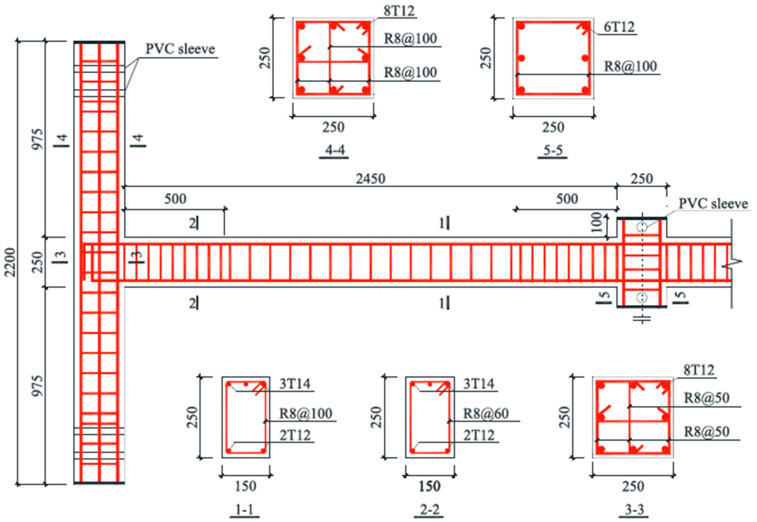
Geometry and reinforcement configuration of the RC-FRS beam–column sub-assemblage used for baseline model validation. The schematic shows the member dimensions, reinforcement layout, stirrup spacing, section details, and PVC sleeve locations of the full-restraint conventional RC specimen. PVC denotes polyvinyl chloride; the PVC sleeves were used in the reference specimen to create unbonded reinforcement regions and allow catenary-action development. All dimensions are given in mm.

The macroscale analysis adopts a controlled two-phase methodology designed to isolate the unique contribution of auxetic metamaterials to progressive collapse resistance. In the first phase, conventional RC beam–column sub-assemblages representing three distinct structural configurations (RC-FRS, RC-FRM, and RC-FRB) are modeled without metamaterial inclusions. These baseline models are validated against established experimental data [30,31] by comparing numerical and experimental load–displacement responses, as detailed in Section 3. This validation establishes a reliable computational benchmark of conventional structural behaviour. The experimental setup adopted in the reference studies [30,31] corresponds to a column-removal scenario for RC beam–column sub-assemblages. In these tests, the sub-assemblage was supported at the column ends, while a vertical displacement-controlled load was applied at the location of the removed column. The displacement was increased gradually under quasi-static loading to capture the complete structural response, including the initial elastic stage, yielding and plastic deformation, post-peak softening, and final failure. During loading, the vertical displacement at the removed-column location and the corresponding reaction force were recorded to obtain the load–displacement curves used for model validation. The reinforcement layout, support conditions, and loading configuration of the reference specimen with auxetic metamaterials inserted at the middle joint region, where large rotation and high tensile demand develop during the column-removal response are shown in Figure 3. It is worth noting that the construction details shown in Figure 3 were adopted from the reference experimental studies [23,24] to ensure direct comparison between the numerical and experimental responses. These details were therefore not newly designed in the present work, but reproduced from the validated test specimens. The progressive-collapse loading condition was modeled as a column-removal scenario, consistent with the alternate-load-path concept used in UFC 4-023-03 for progressive collapse assessment [28].

In the second phase, auxetic metamaterial inserts are introduced into the validated baseline models as the only structural modification. The inserts are positioned in critical joint regions identified from the baseline stress and damage fields. This placement allows the auxetic mechanism to engage during plastic hinge formation and catenary action development. Therefore, comparisons between the RC- and Aux- models are used to evaluate the predicted effect of the auxetic insert while keeping the remaining modeling assumptions unchanged.

Three structural configurations are considered in this study. The RC-FRS/Aux-FRS pair represents the full-restraint frame configuration. The RC-FRM/Aux-FRM pair represents the frame configuration with seismic detailing. The RC-FRB/Aux-FRB pair represents the exterior frame configuration. This paired structure ensures that each auxetic-enhanced model is compared only with its corresponding validated conventional RC baseline.

To avoid ambiguity between the experimentally validated conventional RC specimens and the numerically predicted auxetic-enhanced configurations, a revised naming convention is used throughout the manuscript. The prefix RC- denotes conventional RC specimens adopted from the reference experiments and used for baseline model validation, whereas the prefix Aux- denotes numerical configurations obtained by embedding auxetic metamaterial inserts into the corresponding validated RC baseline models. Accordingly, RC-FRS, RC-FRM, and RC-FRB refer to the conventional full-restraint, seismic-detailing, and exterior-frame RC specimens, respectively. Their auxetic-enhanced numerical counterparts are denoted as Aux-FRS, Aux-FRM, and Aux-FRB, respectively. The RC specimens do not contain auxetic inserts and use the conventional concrete Poisson’s ratio, while the Aux models contain auxetic inserts with the assigned effective negative Poisson’s ratios described in Section 2.4.3. No physical auxetic-enhanced specimens were tested in this study.

### 2.4. Constitutive Models of Materials

#### 2.4.1. Concrete Damage Plasticity Model

**Figure 4:**
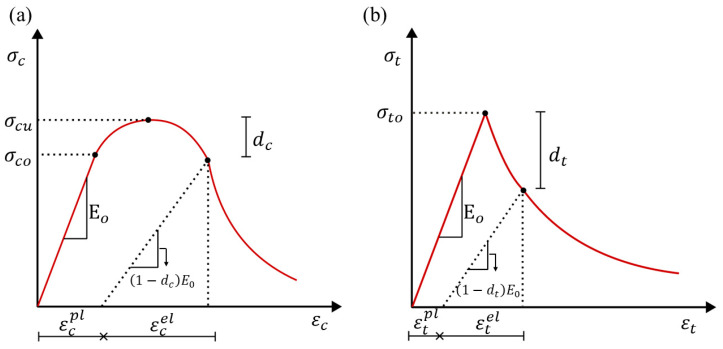
CDP model response under uniaxial loading: (**a**) compressive behaviour with hardening and softening, and (**b**) tensile behaviour with stiffening and fracture-based degradation.

**Figure 5:**
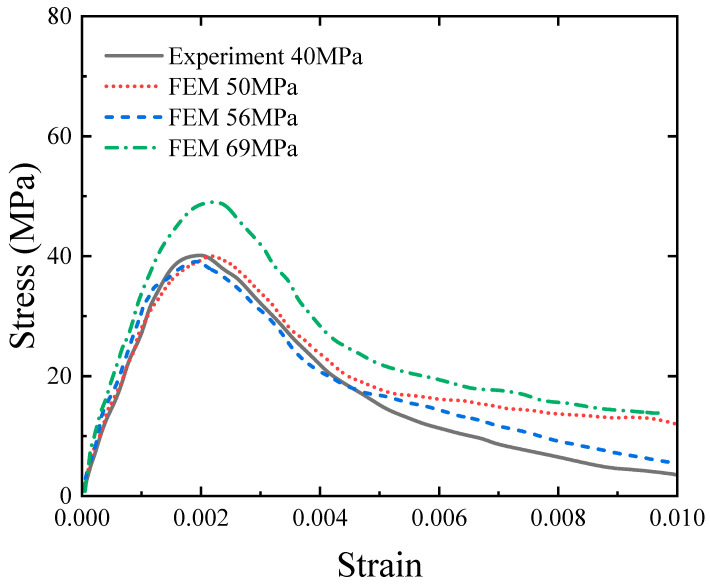
Experimental validation of the concrete constitutive model, demonstrating close agreement between simulated (macroscale and mesoscale) and experimental stress–strain responses under uniaxial compression.

The concrete damage plasticity (CDP) model was first calibrated to provide a reliable baseline response of plain concrete, so that any subsequent improvements in structural performance can be clearly attributed to the auxetic metamaterials rather than to uncertainties in the constitutive law. The model parameters were validated against experimental uniaxial compression data, using the conversion proposed in previous works [32,33] to reproduce the cylindrical test results with the cubic mesoscale specimen adopted in this study. The resulting stress–strain response under uniaxial compression and tension is shown in Figure 4, while Figure 5 compares the calibrated numerical curves (macroscale and mesoscale) with the reference experimental data. For the validation setup, the macroscale model used a Φ150 × 300 mm concrete cylinder subjected to displacement-controlled uniaxial compression, consistent with the reference experiment. The mesoscale model used a 150 × 150 × 150 mm concrete cube under the same loading condition to verify that the heterogeneous aggregate–mortar–ITZ representation could reproduce the converted compressive response. The characteristic mesh sizes were 5 mm for the cylinder model and 1.25 mm for the mesoscale cube, allowing the latter to resolve the aggregate, mortar, and ITZ phases. The adopted CDP model used a flow potential eccentricity of 0.1, a dilation angle of 40°, a biaxial-to-uniaxial compressive strength ratio of 1.16, a second stress invariant ratio of 0.66667, and a viscosity parameter of 0.00001. The elastic response of concrete was defined using a Poisson’s ratio of 0.2, while the Young’s modulus was calculated as *E_0_* = 22,000[(*f_ck_* + 8)/10]^0.3^ MPa. These parameters were used consistently in the macroscale, mesoscale, and sub-model simulations to maintain a unified concrete constitutive framework [34]. This validated constitutive framework is used consistently in all macroscale and sub-model simulations and serves as the reference against which the influence of the auxetic metamaterial inserts, including delayed crushing and more stable cracking patterns, is quantified.

Concrete compressive stress–strain response is weakened when the material appears damaged or degraded. The degradation is defined by the reduction in elastic stiffness of the material [35]. The strain softening behavior is characterized by two damage variables, *d_t_* and *d_c_*, taken between 0 and 1. Therefore, the stress–strain relationships for concrete under uniaxial compression and tension can be described by

(3) $$\sigma_{c}=(1-d_{c})\cdot E_{0}\cdot (\varepsilon_{c}-\varepsilon_{c}^{pl})$$

(4) $$\sigma_{t}=\left(1-d_{t}\right)\cdot E_{0}\cdot \left(\varepsilon_{t}-\varepsilon_{t}^{pl}\right)$$

where $E_{0}$ is the initial (undamaged) elastic stiffness matrix of the material, $\varepsilon_{c}^{pl}$ and $\varepsilon_{t}^{pl}$ are compressive and tensile plastic strain vectors, respectively.

#### 2.4.2. Continuum Damage Mechanics Model for Steel Reinforcement

For steel reinforcement, a combined elastoplastic–damage constitutive model is adopted based on the ductile damage framework in [36], rather than a purely continuum-damage formulation. The plastic response of the reinforcement is described using a von Mises yield criterion with isotropic hardening, which is appropriate for ductile steel under large deformation. The yield strength and ultimate strength are calibrated from the tensile test data reported in the reference studies [30,31,37]. To capture fracture at large deformation, the plasticity model is coupled with a ductile damage initiation criterion. In this criterion, damage initiates when the equivalent plastic strain reaches a critical value that depends on the stress triaxiality. After damage initiation, a displacement-based damage evolution law is used to reduce the effective stiffness and load-carrying capacity of the reinforcement progressively until failure. The reinforcement properties were assigned according to the bar type used in each reference specimen. For RC-FRM and RC-FRS, the T10 longitudinal bars had an elastic modulus of 203 GPa, yield strength of 513 MPa, and ultimate strength of 613 MPa, with fracture strains of 10% and 7%, respectively. The R6 transverse bars had elastic moduli of 220 GPa and 190 GPa, yield strengths of 395 MPa and 248 MPa, and ultimate strengths of 581 MPa and 405 MPa for RC-FRM and RC-FRS, respectively. For RC-FRB, the T12 bar had an elastic modulus of 203 GPa, yield strength of 580 MPa, ultimate strength of 631 MPa, fracture strain of 50%, and displacement at failure of 1 mm. The T10 and R8 bars used elastic moduli of 205 GPa and 210 GPa, yield strengths of 151 MPa and 248 MPa, and ultimate strengths of 398 MPa and 450 MPa, respectively. T10/T12 denote 10/12 mm deformed bars, while R6/R8 denote 6/8 mm round stirrups. The properties marked in the original experimental sources were obtained from tensile tests reported in [30,35].

#### 2.4.3. Constitutive Models for Mesoscale Components of Concrete

The mesoscale model represents concrete as a four-phase composite, where the embedded auxetic metamaterial is integrated as a distinct, active phase alongside aggregate, mortar and the ITZ. This explicit multiphase representation is employed to resolve specific micromechanical interactions, particularly stress transfer and crack bridging, between the auxetic lattice and the surrounding concrete matrix, which cannot be captured explicitly in macroscale analyses. While established models govern the conventional phases [38,39], and a cohesive formulation captures the ITZ as a crack initiation path [23], these representations collectively establish the necessary heterogeneous environment to isolate and quantify the damage-mitigating function of the auxetic metamaterial.

The auxetic metamaterial insert was represented using equivalent continuum properties derived from a re-entrant honeycomb unit-cell homogenization analysis. The adopted unit cell had a re-entrant angle of −30°, an aspect ratio of *h/l* = 1.2, and a wall thickness of 1 mm. The base material was assumed to be polyurethane with *E_s_* = 1.2 GPa and *ν_s_* = 0.35, consistent with polymeric auxetic-lattice applications reported in the literature [40]. Periodic boundary conditions were applied to the unit cell, and the effective properties were calculated from the volume-averaged stress and strain response. This procedure yielded an effective Young’s modulus of 0.35 GPa and an effective Poisson’s ratio of −0.25. The obtained negative Poisson’s ratio falls within the range commonly reported for re-entrant honeycomb structures, whose effective properties depend strongly on cell angle, ligament length, wall thickness, and relative density [41]. In the present study, these values are used as equivalent continuum properties for the numerical feasibility analysis.

Geometrically configured as 50 mm × 50 mm × 150 mm prisms, the inserts occupy a central, critical zone within the 150 mm × 150 mm × 150 mm representative volume shown in Figure 1c. This specific geometry and strategic placement adjacent to the column face within the region of peak stress identified from macroscale analysis are designed to ensure the auxetic mechanism engages precisely during the formation of plastic hinges and the development of catenary action. This targeted activation allows the auxetic confinement mechanism to contribute to stress redistribution and improved joint collapse resistance.

The material properties of the four mesoscale phases were defined separately to reflect their different mechanical roles. The aggregate phase was modeled as the stiffest constituent, with a density of 2500 kg/m^3^, an elastic modulus of 50 GPa, and a Poisson’s ratio of 0.2. The mortar phase was assigned a density of 2200 kg/m^3^, an elastic modulus of 32 GPa, a Poisson’s ratio of 0.2, a compressive strength of 27 MPa, and a tensile strength of 3.7 MPa. The ITZ was modeled as the weaker interfacial phase, with a density of 2200 kg/m^3^, an elastic modulus of 24 GPa, a Poisson’s ratio of 0.2, a compressive strength of 16 MPa, and a tensile strength of 2.8 MPa. The auxetic phase used the homogenized properties described above, with a density of 1600 kg/m^3^, an effective Young’s modulus of 0.35 GPa, an effective Poisson’s ratio of −0.25, and compressive and tensile strengths of 3.2 MPa. These properties were assigned consistently in the mesoscale simulations to represent the aggregate–mortar–ITZ system and the embedded auxetic insert as distinct interacting phases [42].

On the other hand, cohesive elements are adopted for the ITZ to simulate failure and fracture within the cohesive zone, and their properties are listed in Table 2. The mechanical response of the cohesive interface is defined using a bilinear traction–separation law adopted from previous cohesive zone modelling studies [43,44]. In this formulation, the initial elastic stage is represented by a linear increase in nominal traction with separation until the maximum nominal traction is reached, which corresponds to damage initiation. After damage initiation, the interface enters a softening stage, during which the cohesive traction decreases progressively with increasing separation. This degradation represents the gradual loss of load-carrying capacity, microcrack development, and eventual complete separation of the ITZ. Therefore, the bilinear traction–separation relationship provides a simplified but effective representation of crack initiation and propagation in cohesive interface elements.

In the bilinear traction–separation formulation, $t_{n}^{0}$ is the maximum traction force, *δ*^0^_n_ is the corresponding displacement, *δ*^f^_n_ is the displacement at complete separation, and *k*^0^_n_ and *k*_n_ are initial and normal cohesive stiffnesses, respectively. By using the Benzeggagh–Kenane fracture criterion [45,46], the elastic modulus or cohesive stiffness k can be calculated by

(5) $$k_{n}=\frac{t^{0}}{\delta^{0}}$$

The damage initiation criterion used in this scenario is the maximum stress criterion as

(6) $$\max\left\{\frac{\left\langle t_{n}\right\rangle }{t_{n}^{0}},\;\frac{t_{s}}{t_{s}^{0}},\frac{t_{t}}{t_{t}^{0}}\right\}=1$$

where *n*, *s*, and *t* indicate normal, shear and traction, respectively. 〈〉 is the Macaulay bracket, which is defined by

(7) $$\langle t_{n}\rangle =\left\{\begin{array}{cc} t_{n}, & t_{n\;}\geq 0\\ 0, & t_{n}<0 \end{array}\right.$$

The damage evolution law specifies the progression of the damage, stating how the material contact stiffness is degraded. A scalar index *D* is used to specify the overall damage bond, which evolves from 0 to 1, where 0 indicates the state without damage and 1 indicates the complete damage state. The damage variables affecting stress components [44] are defined by

(8) $$t_{n}=\left\{\begin{array}{cc} \left(1-D\right){\bar{t}}_{n}, & {\bar{t}}_{n}\;\geq 0\\ 0, & {\bar{t}}_{n}<0 \end{array}\right.$$

(9) $$t_{s}=\left(1-D\right){\bar{t}}_{s}$$

(10) $$t_{t}=\left(1-D\right){\bar{t}}_{t}$$

#### 2.4.4. Justification for Homogenized Auxetic Insert Properties

The auxetic insert is modeled using homogenized isotropic continuum properties, with *E* = 0.35 GPa and *ν* = −0.25, rather than by explicitly resolving the re-entrant lattice geometry. This simplification is adopted for three reasons. First, the insert is intended to expand laterally under tensile deformation and transfer confinement to the surrounding concrete. This response depends mainly on the effective negative Poisson’s ratio and global stiffness of the insert. The local lattice topology and cell orientation may affect the detailed stress field inside the insert, but they are not expected to qualitatively change the primary confinement mechanism examined in this study. However, the present study does not include a full sensitivity analysis of lattice geometry variations. This limitation is acknowledged, and future experimental validation should verify whether local geometric details alter the predicted mechanism.

Second, the adopted re-entrant honeycomb geometry, with *θ* = −30°, *h/l* = 1.2, and *t* = 1 mm, can be represented by effective continuum properties when several unit cells exist within the insert volume [41]. The insert dimensions, 50 × 50 × 150 mm, contain multiple repeating cells, which supports the use of a homogenized representation for the present structural and sub-model analyses. Third, the loading condition is quasi-static, with a displacement rate of 2 mm/min [30]. Under this loading rate, strain-rate effects in the polyurethane base material are expected to have limited influence on the predicted collapse response. A rate-independent homogenized model is therefore considered adequate for this numerical feasibility study.

This modeling choice remains a simplification. Future work should test fabricated auxetic inserts and explicitly model the lattice geometry to examine the influence of cell orientation, lattice anisotropy, and strain-rate sensitivity. In the present study, however, the homogenized representation is sufficient to evaluate whether the effective lateral expansion of the auxetic phase can modify confinement, stress redistribution, and damage progression near the joint.

### 2.5. Discretization, Interactions, and Boundary Condition

A mesh convergence study was first carried out for the conventional RC baseline models, namely RC-FRS, RC-FRM, and RC-FRB, for which published experimental data are available. The purpose of this study was to select mesh sizes that provide stable load–displacement predictions while maintaining reasonable computational cost. The selected mesh strategy was then retained for the corresponding auxetic-enhanced numerical models, namely Aux-FRS, Aux-FRM, and Aux-FRB, so that the comparison between conventional and auxetic-enhanced configurations would not be affected by changes in discretization.

**Figure 6:**
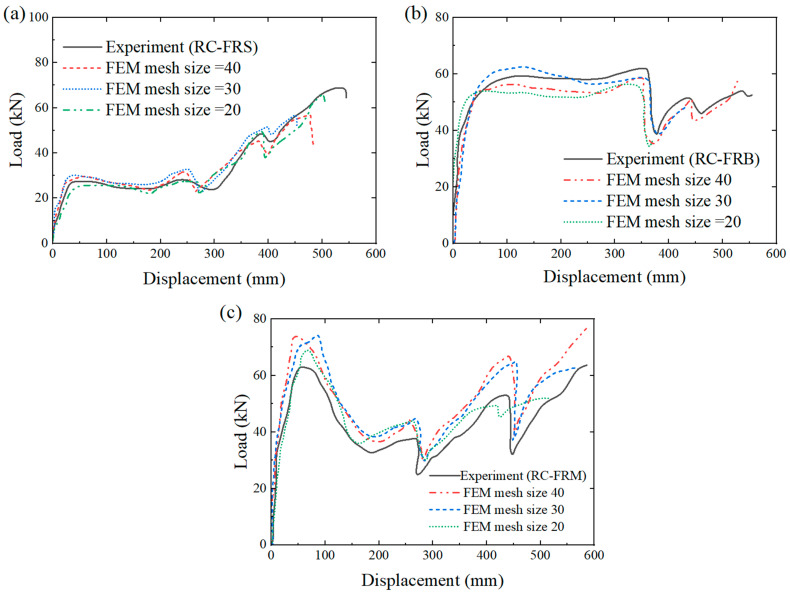
Mesh sensitivity analysis demonstrating numerical convergence for the three beam–column sub-assemblages: (**a**) RC-FRS, (**b**) RC-FRB, and (**c**) RC-FRM.

Figure 6 shows the influence of mesh size on the load–displacement response of the three conventional RC baseline models. For RC-FRS, further mesh refinement below 40 mm produced only minor changes in the global response. For RC-FRM, a mesh size of 30 mm provided stable prediction of the peak load and post-peak response. For RC-FRB, a mesh size of 20 mm was required to capture the stronger localization near the exterior joint. Based on these results, the selected mesh sizes were 40 mm for RC-FRS, 30 mm for RC-FRM, and 20 mm for RC-FRB. The experimental curves shown in Figure 6 correspond only to conventional RC specimens from the literature and are used to validate the baseline numerical framework. No experimental validation of auxetic-enhanced specimens is claimed in this study.

After the baseline mesh sizes were selected, the same global mesh strategy was applied to the corresponding auxetic-enhanced models. In regions containing auxetic inserts, local mesh refinement was introduced to better resolve the stress transfer between the insert, surrounding concrete, and reinforcement. For example, in the Aux-FRM model, a fine mesh of 5 mm was used in the critical joint region containing the auxetic insert, while a coarser 30 mm mesh was maintained in regions away from the joint. This local refinement improves the resolution of strain gradients near the insert without increasing the computational cost of the entire model. The reinforcement mesh was refined consistently with the surrounding concrete mesh to maintain compatible force transfer in the critical joint region.

## 3. Steel Reinforcement Response and Comparative Analysis

### 3.1. Reinforcement Strain Response at Critical Structural Locations

**Figure 7:**
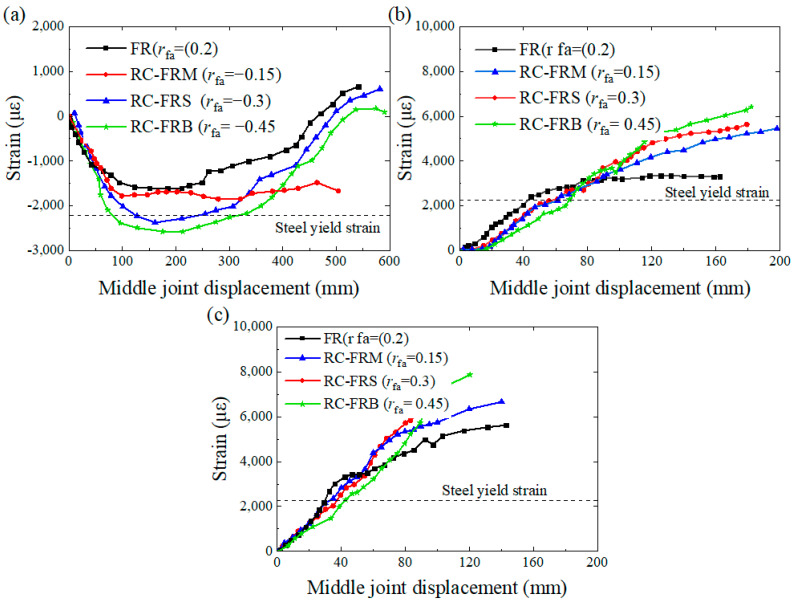
Critical regions isolated for multiscale analysis: (**a**) beam–column joint, (**b**) beam bottom in tension, and (**c**) column base.

The strain response of longitudinal reinforcement was evaluated at three critical locations during progressive collapse: the beam–column joint, the beam bottom in tension, and the column base, as shown in Figure 7. These locations were selected because they govern joint rotation, tensile membrane action, and force redistribution during the column-removal response. The comparison between the conventional RC baseline models and the auxetic-enhanced numerical models indicates that the auxetic insert modifies the predicted strain distribution near the critical joint region.

The predicted change is mainly associated with the lateral expansion of the auxetic insert under tensile deformation. Because of its negative Poisson’s ratio, the insert expands into the surrounding concrete and generates localized confinement near the joint. This confinement may reduce tensile stress concentration and delay the development of severe local damage. In the joint region shown in Figure 7a, the Aux-FRB model predicts a reduction in representative compressive strain from −2200 µε in the corresponding RC-FRB baseline model to −1100 µε, corresponding to an approximately 50% reduction. The tensile strain capacity also increases from 800 µε to 2000 µε, suggesting a greater ability of the joint region to accommodate tensile deformation in the numerical model.

The beam-bottom response in Figure 7b further shows that the auxetic-enhanced models predict larger tensile strains than the corresponding RC baseline models. This result suggests that the auxetic insert may help spread deformation over a longer reinforcement region, which could support tensile membrane and catenary-action development. Meanwhile, the column-base response in Figure 7c shows a more gradual strain evolution in the auxetic-enhanced models, suggesting a more stable load-transfer process during large displacement.

The reinforcement strain response can be divided into three stages: elastic response, strain hardening, and strain localization. In the conventional RC baseline models, strain localization begins at approximately 150 mm displacement. In the auxetic-enhanced numerical models, the onset of severe strain localization is predicted to occur at approximately 220 mm displacement. This corresponds to an estimated 47% delay in critical strain localization. Therefore, the predicted ductility improvement is attributed to confinement-induced strain redistribution near the joint, rather than to an increase in the intrinsic strength of the reinforcement.

### 3.2. Systematic Performance Enhancement

Quantitative strain data presented in Table 3 indicate that the simulated auxetic reinforcement modifies the strain response along the critical load path, from the joint region to the column base. The predicted changes are generally consistent with the assigned negative Poisson’s ratio values used for the auxetic inserts.

The data suggest a coordinated change in the numerical response. In the beam–column joint, the predicted peak compressive strain decreases by up to 55%, while the tensile strain capacity increases by up to 150%. These results suggest a shift from localized crushing toward a more distributed, confinement-influenced damage pattern. The simulated auxetic reinforcement provides these predicted improvements through lateral expansion and localized confinement, rather than through increased reinforcement strength or delayed yielding. In the beam region, the models predict up to 100% higher tensile strain capacity, which may support catenary-action development. At the column base, the predicted strain capacity increases by up to 45%, indicating improved load transfer in the numerical model.

### 3.3. Stress Distribution in Steel Reinforcement: Comparative Analysis with and Without Auxetic Metamaterial

**Figure 8:**
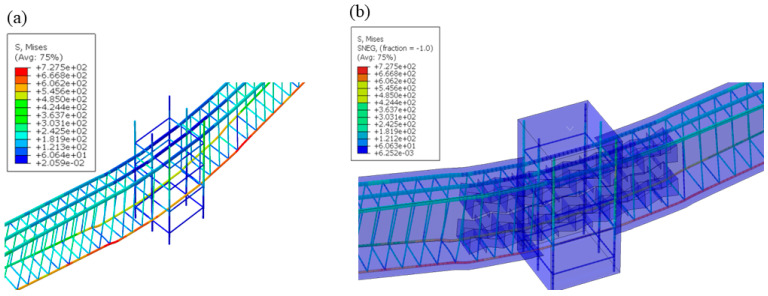
Von Mises stress distribution in steel reinforcement: (**a**) conventional frame without metamaterial showing severe stress concentrations (max stress 350 MPa at joint regions), and (**b**) auxetic-enhanced frame with metamaterial showing uniform stress distribution.

Figure 8 compares the von Mises stress distribution in the steel reinforcement for the conventional and auxetic-enhanced configurations. In the conventional frame, the reinforcement stress is concentrated near the beam–column interface, with a maximum von Mises stress of approximately 350 MPa. This stress pattern indicates that load transfer occurs through a short-localized region, causing only a limited portion of the reinforcement to participate effectively in resisting the applied deformation. In this case, less than 15% of the reinforcement length carries stresses above 400 MPa, which is consistent with early strain localization near the joint.

In the auxetic-enhanced configuration, the maximum local von Mises stress reaches approximately 728 MPa near the critical joint region. This value exceeds the ultimate strength and should therefore not be interpreted as an allowable uniform stress state in the reinforcement. Instead, it represents a localized peak in the numerical stress contour. Accordingly, the auxetic insert should not be understood as reducing the maximum von Mises stress or delaying steel yielding at the location of this peak. Its main effect is to modify the spatial distribution of stress along the reinforcement.

This redistribution is shown in the stress-profile comparison in Figure 8b. In the conventional frame, stresses above 400 MPa are confined to less than 15% of the reinforcement length, indicating severe stress localization. By contrast, in the auxetic-enhanced frame, stresses above 400 MPa extend over approximately 60–70% of the reinforcement length. This result suggests that a larger portion of the reinforcement participates in resisting the imposed deformation. Although the local peak stress is higher, the stress-transfer region is wider, and the response is less dependent on a single critical section.

Therefore, the improved response of the auxetic-enhanced configuration is attributed to stress redistribution and delayed critical strain localization, rather than to a reduction in peak stress or delayed yielding of the reinforcement. The reinforcement response should be interpreted together with the stress distribution along the bar and the strain evolution shown in Figure 7. Based on these results, the auxetic-enhanced configuration supports a more distributed load-transfer mechanism and a more stable transition from flexural action to catenary action during progressive collapse.

## 4. FE Simulation Results and Discussions

The integration of auxetic metamaterials alters the mechanical response of the beam–column joint by introducing an active confinement mechanism that contributes to energy dissipation and delayed damage progression. While traditional seismic detailing (RC-FRM) relies on static confinement, the negative Poisson’s ratio of the auxetic inserts triggers a dynamic confining pressure that scales with the loading demand. As the joint undergoes extreme rotation, the lateral expansion of the metamaterial counters tensile splitting stresses within the concrete core, preserving the effective cross-sectional integrity of the compression zone.

### 4.1. Full Restraint-Conventional Detailing (RC-FRS)

**Figure 9:**
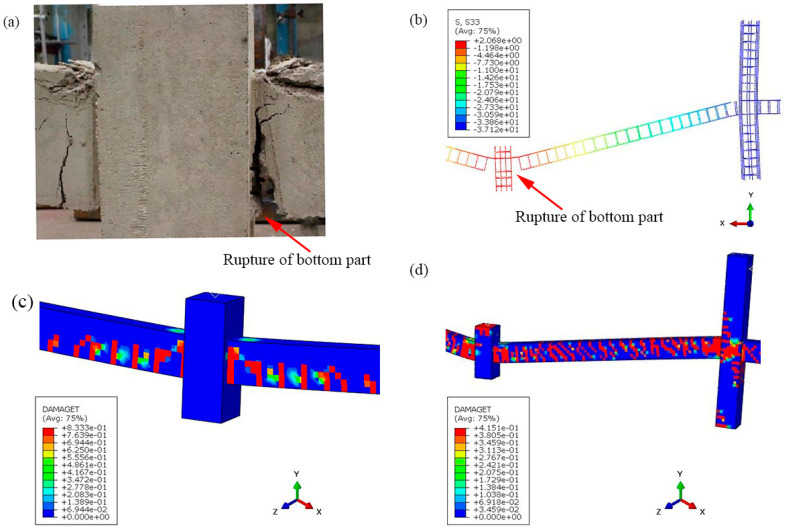
Experimental and numerical validation of failure mechanisms: (**a**) experimental crack pattern, (**b**) bottom bar rupture, (**c**) concrete compressive damage, and (**d**) concrete tensile damage in specimen RC-FRS.

As shown in Figure 9, the numerical model reproduces the main failure characteristics observed in the reference experiment, including damage concentration near the beam–column joint and the subsequent development of the collapse mechanism. The integration of auxetic metamaterials fundamentally redefines the collapse resistance of the baseline RC-FRS connection by activating a deformation-dependent confinement mechanism. This performance shift originates from the insert’s negative Poisson’s ratio, where lateral expansion under axial tensile strain generates a localized confining pressure that directly impedes concrete crushing within the plastic hinge zone.

The structural consequence of this active confinement is an 8.7% delay in the critical failure transition, with the first significant load reduction deferred until 250 mm of displacement. This delay is primarily attributed to the auxetic mechanism, which enables the configuration to sustain a reaction force of 40 kN during the catenary phase, representing a clear improvement in load-carrying capacity and ductility over the unmodified RC-FRS frame. By actively governing internal stress redistribution and suppressing damage progression at the microstructural level, the embedded metamaterial ensures a prolonged, stable load path that conventional detailing cannot achieve.

The load–deformation response was evaluated at three behavioral stages: compressive arch action (CAA), catenary-action initiation, and catenary-action domination. For the conventional RC-FRS specimen, the experimental response reached approximately 25 kN at 55 mm during CAA, 17.5 kN at 252.5 mm when catenary action initiated, and 66 kN at 540 mm when catenary action dominated. The corresponding RC-FRS numerical model predicted 28.8 kN at 72 mm, 15 kN at 249 mm, and 40 kN at 455 mm, respectively. These results show that the model captures the main response stages, although differences remain in the final catenary-dominated stage because reinforcement fracture, bond degradation, and local damage accumulation are simplified in the numerical model. For the other reference configurations, the numerical predictions also followed the experimental response trends. The RC-FRB model predicted 29.7 kN at 53 mm, 24.3 kN at 251.5 mm, and 57 kN at 465 mm, while the RC-FRM model predicted 57 kN at 110 mm, 25.5 kN at 395 mm, and 47 kN at 550 mm, compared with the corresponding RC-FRM experimental values of 54 kN at 95 mm, 45 kN at 400 mm, and 62 kN at 553 mm. Overall, the comparison supports the use of the validated baseline models for subsequent numerical evaluation of auxetic-enhanced configurations. After baseline validation, the auxetic insert was introduced into the corresponding numerical model to assess its predicted influence on collapse resistance. The auxetic mechanism is activated by lateral expansion under tensile deformation, which generates localized confinement in the joint region. In the auxetic-enhanced configuration, the first significant load reduction was delayed to approximately 250 mm, and the model sustained about 40 kN during the catenary phase. These values should be interpreted as numerical predictions rather than experimentally measured improvements.

### 4.2. Full Restraint- Seismic Detailing (RC-FRM)

**Figure 10:**
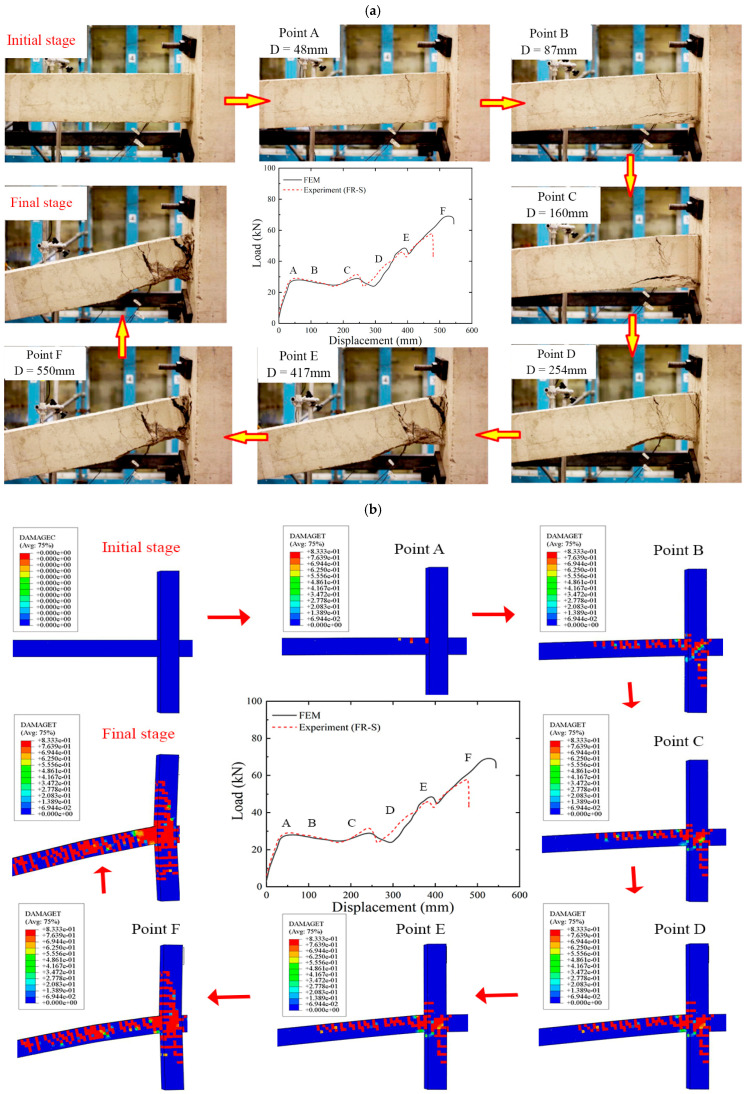
Mechanism of auxetic metamaterial action in (**a**) experimental study, and (**b**) numerical prediction of the present study: Sequential crack formation and stress redistribution at the left beam end of specimen RC-FRS, demonstrating enhanced post-yield stiffness and suppressed concrete spalling through active confinement.

Figure 10 compares the experimental crack pattern of the conventional RC-FRM specimen with the numerical damage distributions obtained from the corresponding baseline model and the auxetic-enhanced Aux-FRM model. The experimental result is used to validate the conventional RC baseline model, whereas the Aux-FRM result represents a numerical prediction derived from the validated baseline. The comparison shows that the baseline numerical model captures the dominant cracking region and the overall failure mode, although local crack details may differ because the simulation represents concrete cracking through continuum damage variables rather than directly resolved discrete cracks.

In the auxetic-enhanced Aux-FRM model, the insert modifies the predicted damage pattern near the beam end. Under tensile deformation, the negative Poisson’s ratio of the auxetic insert drives lateral expansion and produces a localized confinement effect in the surrounding concrete. This confinement reduces tensile splitting near the joint and helps maintain the compression zone during large rotation. As a result, the numerical damage field shows a more distributed cracking pattern and less localized cover damage than the corresponding conventional RC-FRM baseline. The numerical comparison indicates that the Aux-FRM configuration shows a predicted 25% increase in deformation capacity compared with the corresponding RC-FRM baseline model. This predicted improvement is attributed to the confinement-induced redistribution of damage and the delayed development of severe localization near the joint. Because no physical auxetic-enhanced specimen was tested in the present study, this value should be interpreted as a numerical prediction that requires future experimental validation.

Although the present numerical results are compared with the experimentally observed crack patterns reported in the reference tests, the comparison remains primarily qualitative because full-field experimental strain data were not available. Digital image correlation (DIC) would provide a more quantitative basis for experimental–numerical validation by measuring surface displacement and strain fields during loading. Such data could be used to compare the predicted and measured crack initiation locations, strain localization bands, crack opening evolution, and damage progression. Recent studies have demonstrated the usefulness of DIC for capturing shear sliding behavior in strengthened masonry, identifying instability and deformation modes in composite shells, and calibrating finite element material parameters from full-field measurements [47,48,49]. Therefore, future experimental studies on auxetic-enhanced RC joints should incorporate DIC measurements to validate the predicted damage-mitigation mechanism and to quantify the transition from localized cracking to distributed damage.

### 4.3. Exterior RC Frame with Seismic Detailing (RC-FRB)

**Figure 11:**
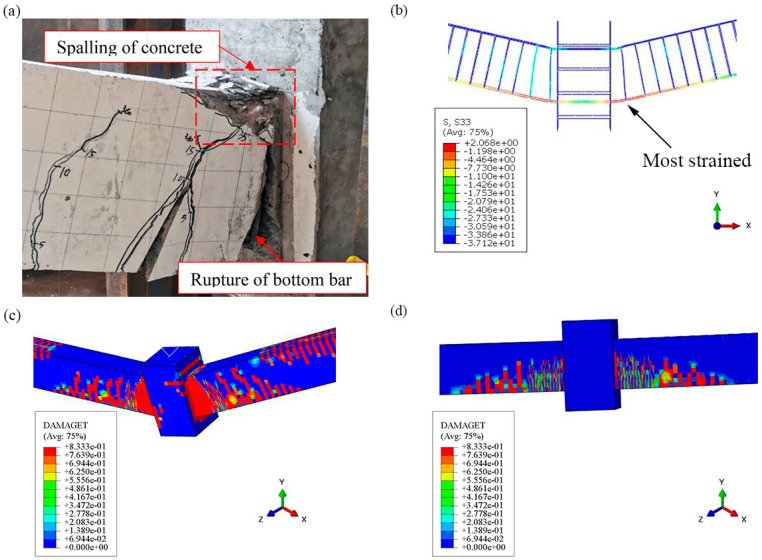
Validation of failure mechanisms in exterior RC frame (RC-FRB): comparisons of (**a**) experimental crack pattern with (**b**) numerical simulations of reinforcement yielding, (**c**) concrete compressive crushing, and (**d**) concrete tensile damage.

In the exterior frame configuration (RC-FRB), the numerical model was first validated against the reference experimental response before introducing the auxetic-enhanced configuration. As shown in Figure 11a, the experimental specimen exhibited concrete spalling near the exterior joint and rupture of the bottom reinforcement. The numerical model reproduces the main failure characteristics of this specimen. Figure 11b shows that the reinforcement reaches the highest strain near the joint region, while Figure 11c,d shows that compressive crushing and tensile damage develop in the same critical region. This agreement indicates that the model captures the dominant failure mechanism of the exterior RC frame, including localized concrete damage and high tensile demand in the bottom reinforcement.

**Figure 12:**
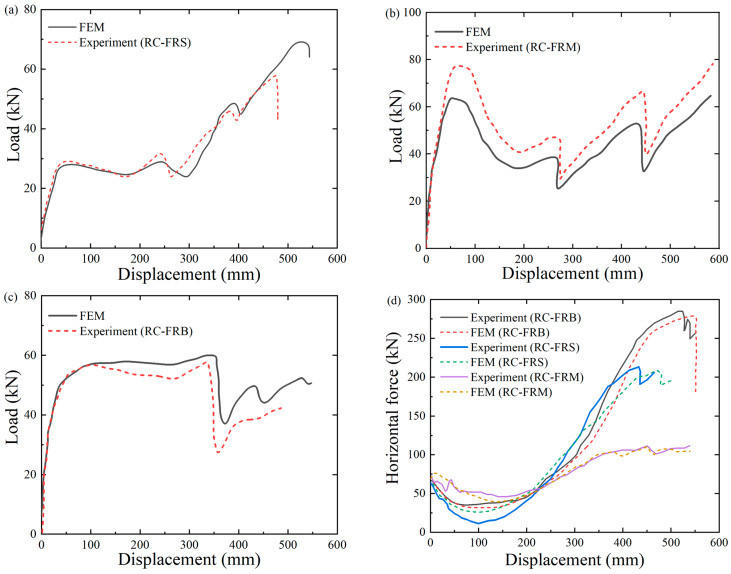
Comparison between numerical and experimental global responses: (**a**–**c**) vertical load–displacement behaviour for specimens RC-FRS, RC-FRB, and RC-FRM; and (**d**) ductility comparison showing deformation capacity for each configuration.

Figure 12 further compares the numerical and experimental global responses of the reference specimens. In Figure 12a, the RC-FRS specimen shows good agreement between the numerical and experimental load–displacement curves through the elastic stage and most of the nonlinear response. The model also captures the post-yield load redistribution and the later increase in resistance associated with catenary action. The remaining difference near the final displacement may be related to local fracture, reinforcement rupture, and damage accumulation, which are difficult to reproduce exactly in a continuum FE model. For the RC-FRM specimen in Figure 12b, the numerical and experimental curves agree well in the initial elastic stage but diverge after cracking and plastic deformation develop. The experimental response shows a higher resistance during part of the nonlinear regime, whereas the numerical model predicts a lower intermediate load level. This difference may result from local confinement, reinforcement anchorage, bond-slip behavior, and material variability in the test specimen, which are simplified in the present model. Since the model uses idealized concrete damage plasticity parameters and simplified steel–concrete interaction, exact agreement in the post-cracking regime is not expected. For the RC-FRB specimen in Figure 12c, the numerical model slightly overestimates the experimental response after the major load drop. This overestimation may be related to the idealized representation of residual load transfer after damage initiation. In the experiment, concrete spalling, local bond degradation, and reinforcement slip can reduce the effective stiffness and load-carrying capacity after cracking. These local mechanisms are represented only approximately in the macroscale FE model. Nevertheless, the model captures the main behavioral stages, including the initial elastic response, nonlinear stiffness change, major load drop, post-peak response, and residual resistance.

Figure 12d compares the horizontal force response and deformation capacity of different configurations. The comparison shows that the numerical model reproduces the overall deformation trend and the relative differences among the reference specimens. Therefore, despite local discrepancies in the nonlinear regime, the model is considered adequate for evaluating the relative influence of auxetic inserts on collapse resistance. After validating the baseline response, auxetic metamaterial inserts were introduced into the critical joint region of the RC-FRB model. The auxetic system provides a deformation-dependent confinement mechanism through lateral expansion under tensile deformation. This mechanism improves local stress transfer, delays severe strain localization, and supports a more stable transition toward catenary action. These results should be interpreted as numerical predictions that require future experimental validation.

### 4.4. Local Response from the Sub-Modeling Analysis

The sub-modelling technique resolves the micromechanical mechanisms governing progressive collapse resistance within metamaterial-embedded regions. Critical joint zones are extracted from the validated macroscale models (Section 3) for high-resolution analysis. Displacement boundary conditions, interpolated from the parent model solutions, ensure kinematic consistency while allowing the refined mesh to capture localized phenomena homogenized in the macroscopic simulation.

**Figure 13:**
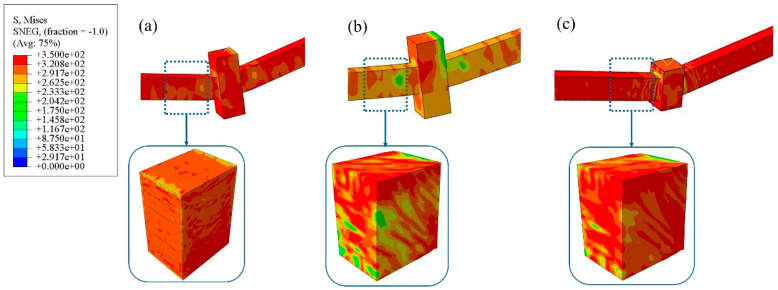
Enhanced resolution of principal tensile stress: Sub-model versus macroscale predictions for (**a**) RC-FRM, (**b**) RC-FRS, and (**c**) RC-FRB specimens, demonstrating superior localization of stress concentrations.

This high-fidelity analysis in Figure 13 quantifies the fundamental stress mitigation mechanisms enabled by auxetic inserts. The sub-model reveals that lateral expansion of the auxetic lattice under tensile loading generates a localized confining stress field within the concrete matrix, with compressive pressures reaching up to 5 MPa, which is sufficient to actively counteract principal tensile stresses. This mechanism induces a systematic transition from localized macrocracking to diffuse microcracking patterns, suggesting a damage-control functionality of the metamaterial in the numerical model.

As summarized by the stress and strain indicators, the sub-model provides a more localized description of the critical joint response than the macroscale model. For RC-FRM, the macroscale model predicts an S22 stress and principal tensile stress of 3.397 MPa, with PEEQ and PEEQT values of 0.372 and 0.593, respectively. The corresponding sub-model gives lower stresses of 1.991 MPa, with PEEQ and PEEQT reduced to 0.310 and 0.292. For RC-FRS, the macroscale model predicts S22 and principal tensile stresses of 2.232 MPa and 3.032 MPa, while the sub-model predicts 1.178 MPa and 1.648 MPa. In this case, the sub-model gives higher localized plastic strain values, with PEEQ and PEEQT increasing from 0.120 and 0.100 to 0.296 and 0.345, respectively. For RC-FRB, the sub-model reduces the predicted S22 and principal tensile stress from 1.856 MPa and 2.778 MPa to 0.801 MPa and 1.393 MPa, while the localized strain indicators increase from 0.091 and 0.955 to 0.380 and 2.430. These differences show that the sub-model does not simply reproduce the macroscale response; instead, it resolves localized stress relaxation and strain concentration in the critical joint region, which are averaged out in the macroscale model.

This analysis provides critical mechanistic verification within the multiscale framework by establishing the physical link between the global performance enhancements observed in Section 3 and their micromechanical origins. The results confirm that the transition from brittle to ductile failure modes originates from the auxetic lattice’s ability to generate confining pressures and redistribute strain, offering fundamental insights for optimizing metamaterial architecture in collapse-resistant structural design.

**Figure 14:**
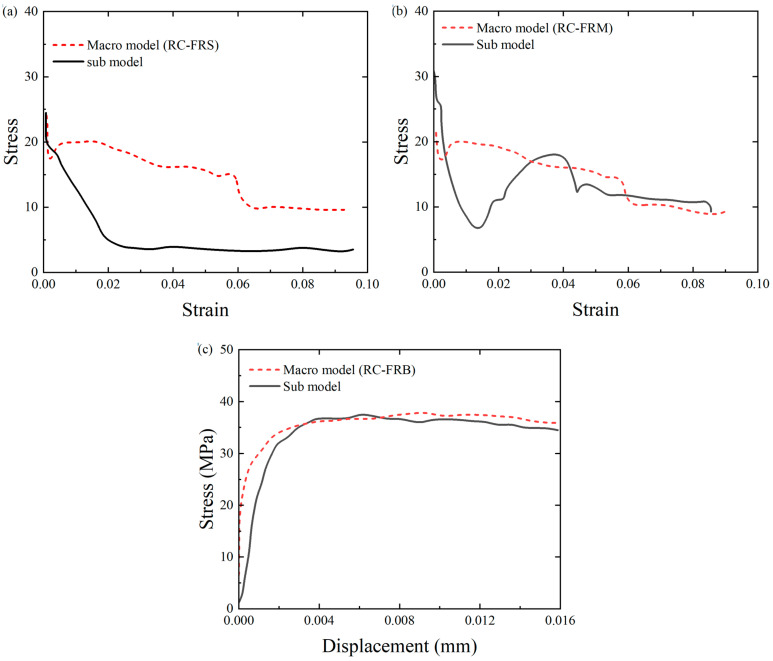
Local stress–strain response at critical joint regions: Comparison between macroscale and sub-model predictions demonstrating enhanced resolution of constitutive behaviour for (**a**) RC-FRS, (**b**) RC-FRM, and (**c**) RC-FRB specimens.

Figure 14 presents a comparative stress–strain analysis derived from high-fidelity sub-modelling, a methodology specifically designed to deconstruct the micromechanical mechanisms through which embedded auxetic metamaterials enhance progressive collapse resistance. This analysis transcends conventional validation; it is a targeted investigation into the novel internal force-redistribution pathways uniquely enabled by the auxetic phase. The sub-model extracts the critical joint region from validated macroscale frameworks, maintaining kinematically consistent boundary conditions while introducing the auxetic insert as the sole variable, thereby isolating its mechanical contribution.

The results reveal a fundamental divergence in stress evolution between modelling scales. The macroscale simulation, while globally accurate, inherently homogenizes stress concentrations. In contrast, the sub-model explicitly resolves the stress-redistribution mechanism activated by the auxetic lattice, providing direct visualization of how lateral expansion under tensile strain transfers principal tensile stresses away from critical interfaces and into the energy-absorbing metamaterial network. This micromechanical intervention has direct structural consequences. The expanding auxetic phase generates a compressive self-confinement effect within the concrete matrix, which delays micro-crack coalescence and stabilizes the post-yield response. Consequently, the internal load path is optimized, directly explaining the enhanced energy dissipation and deformation capacity predicted for metamaterial-enhanced configurations. Thus, this sub-modelling analysis provides the foundational micromechanical evidence that links architectural material design to systemic structural ductility, offering a mechanistic basis for the design of collapse-resistant concrete structures through targeted metamaterial integration.

### 4.5. Plastic Damage of Concrete at Middle Joint

Comparative analysis of damage initiation and propagation between macroscale and sub-model approaches provides crucial insights into global structural behaviour and localized failure processes during progressive collapse. As evidenced in Figure 13, principal tensile stresses computed by macroscale models for RC-FRS and RC-FRM configurations at the beam–column interface substantially exceed those predicted by corresponding sub-models. This discrepancy reflects an inherent limitation of macroscale modelling: the homogenization approach necessarily smears local stress concentrations, whereas the sub-model reveals how these stresses are mitigated through microstructural interactions with the embedded metamaterial. The sub-model demonstrates superior capability in simulating crack growth and interaction patterns, showing remarkable consistency with experimental crack propagation observations. A novel mechanistic insight emerging from this analysis is the clear visualization of damage bifurcation phenomena. Rather than permitting unimpeded propagation of single critical cracks, auxetic inserts actively force damage to branch into multiple smaller micro-cracks. This bifurcation mechanism substantially increases strain energy dissipation through creation of more tortuous crack paths, thereby elevating the effective fracture energy of the composite material and significantly delaying development of continuous failure planes.

**Figure 15:**
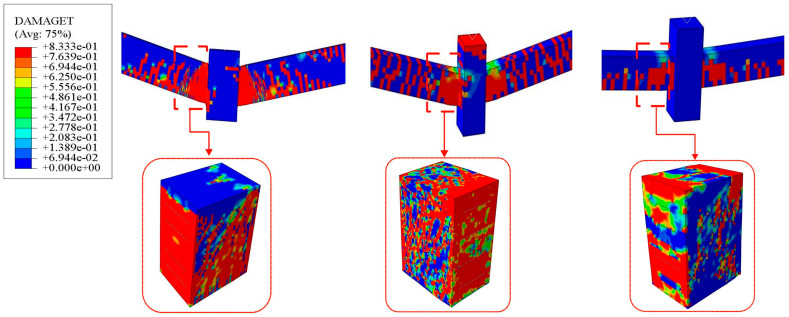
Enhanced damage localization: Comparative plastic damage distribution between macroscale and sub-model simulations for specimens RC-FRM, RC-FRS, and RC-FRB.

Analysis of tensile stress trajectories confirms they typically follow paths of least resistance during crack propagation, with conventional reinforcement providing supplementary crack initiation resistance and width control. The refined sub-models, as illustrated in Figure 15, consistently demonstrate reduced crack widths and extended crack trajectories, accurately capturing flexural cracks exceeding concrete’s tensile capacity while aligning with experimental evidence. The observed crack width reduction in the numerical model is consistent with the predicted auxetic mechanism, wherein metamaterial lateral expansion under tensile strain actively imposes crack-closing pressure against fracture surfaces. This self-confinement effect crucially preserves shear friction and aggregate interlock across crack interfaces, which are essential mechanisms for maintaining post-cracking structural integrity and ductility.

Damage progression further suggests the predicted effect of the auxetic insert. As shown in Table 4, the maximum damage factor decreases from 0.92 in RC-FRM to 0.83 in Aux-FRM. The same trend appears at the other response stages, including peak load, strain localization onset, and failure. These lower damage values indicate that the auxetic-enhanced model delays damage growth at the middle joint. The maximum value of 0.83 still shows notable plastic degradation at the beam–column interface, but it remains below the level observed in the conventional model. This result suggests that the auxetic insert helps spread damage more gradually, rather than allowing rapid localization at one critical region.

The sub-model also provides a clearer view of local stress and damage evolution. It captures stress redistribution and damage concentration near the middle joint with higher spatial resolution than the macroscale model. These results suggest that the auxetic insert improves local damage tolerance in the numerical model by reducing severe localization and promoting a more distributed damage pattern. This mechanism may complement conventional reinforcement detailing, but it should be confirmed through future tests on auxetic-enhanced RC specimens.

From a strengthening perspective, the proposed auxetic insert should also be distinguished from conventional retrofitting or enhancement techniques. Compared with established strengthening and retrofitting techniques such as engineered cementitious composite (ECC) [50] and fiber reinforced polymers (FRP) [51], the proposed auxetic metamaterial insert provides a different damage-mitigation mechanism. FRP systems mainly improve structural resistance through passive external confinement and tensile strengthening. However, their confinement effect depends on the deformation of the surrounding concrete and does not actively change with the internal strain demand of the joint. In contrast, the negative Poisson’s ratio behavior of the auxetic insert allows it to expand laterally under tensile deformation, thereby generating a strain-dependent local confinement effect in the critical joint region. ECC improves ductility through strain-hardening behavior and distributed microcracking, but its use generally requires replacement or modification of the cementitious material in the strengthened region. The auxetic insert, by comparison, can be locally placed within selected high-demand zones without altering the bulk concrete composition. Nevertheless, the present study should be regarded as a numerical feasibility investigation. Future work should further examine hybrid strengthening strategies, such as combining auxetic inserts with FRP wrapping or ECC jacketing, to determine whether these approaches can provide complementary improvements in ductility, confinement, and collapse resistance.

## 5. Discussion of Failure Mechanism Using Mesoscale Model

This investigation employs mesoscale modelling to examine the failure mechanisms of concrete under progressive collapse conditions, with particular focus on how embedded auxetic metamaterials actively alter traditional damage progression. While steel reinforcement provides fundamental resistance through elastoplastic behaviour, the mesoscale approach enables a detailed examination of how the auxetic phase interacts with AVF and ITZ properties to govern crack initiation and propagation.

A key contribution of this study is the ability to relate the global structural enhancements quantified in Section 3 to distinct micromechanical contributions from each material phase. Whereas macroscale models predicted global load–displacement response, the mesoscale representation illustrates the predicted underlying physics: the auxetic metamaterial actively reorganizes internal stress fields and creates novel damage pathway.

The mesoscale model was extracted from stress-critical joint regions identified in the sub-model analysis (Figure 1c) and was assigned the material properties mentioned in Section 2.4.3 and listed in Table 2. The mesoscale model demonstrates that the auxetic insert does not merely coexist with the concrete matrix but actively interacts with the aggregate–mortar–ITZ system. This interaction manifests as fundamental redirection of crack paths and enhanced energy absorption at interfaces traditionally representing the composite’s weakest links.

This research advances beyond prior mesoscale investigations of material properties by indicating that architectural inclusions may be designed to actively compensate for inherent weaknesses. The findings, supported by stress–strain responses and damage evolution sequences, suggest a shift in perspective from treating concrete as a passively heterogeneous material toward designing it as an actively interacting multiphase system. The indicated ability to control damage evolution through metamaterial integration, suggested by a predicted 25% greater energy dissipation and stabilized post-peak ductility, may offer new possibilities for designing concrete structures with engineered failure modes and enhanced collapse resistance. This means that the findings of this study could be extended to the design and analysis of other types of structures [52,53,54,55,56,57,58,59], such as walls, beams and tunnels, where the incorporation of auxetic metamaterials may improve structural performance under extreme conditions.

### 5.1. Mesh Sensitivity Study

To ensure the reliability of mesoscale simulations specifically designed to resolve the novel deformation mechanisms of embedded auxetic metamaterials, a comprehensive mesh sensitivity analysis was conducted as performed previously by the authors [42,60]. Models with realistic polyhedral aggregates, 40% AVF, aggregate sizes of 4.75–19 mm, and a centrally placed auxetic insert were evaluated using four mesh sizes: 0.75, 1.00, 1.25, and 1.50 mm. This convergence study is particularly important for capturing the strain-gradient-dependent auxetic expansion and its interaction with the heterogeneous concrete matrix, which requires finer resolution than conventional concrete modelling. The corresponding CPU times were 23,858 s for the 0.75 mm mesh, 20,462 s for the 1.00 mm mesh, 10,216 s for the 1.25 mm mesh, and 3494 s for the 1.50 mm mesh. Based on the balance between numerical stability and computational efficiency, the 1.25 mm mesh was selected for the subsequent mesoscale simulations [42].

**Figure 16:**
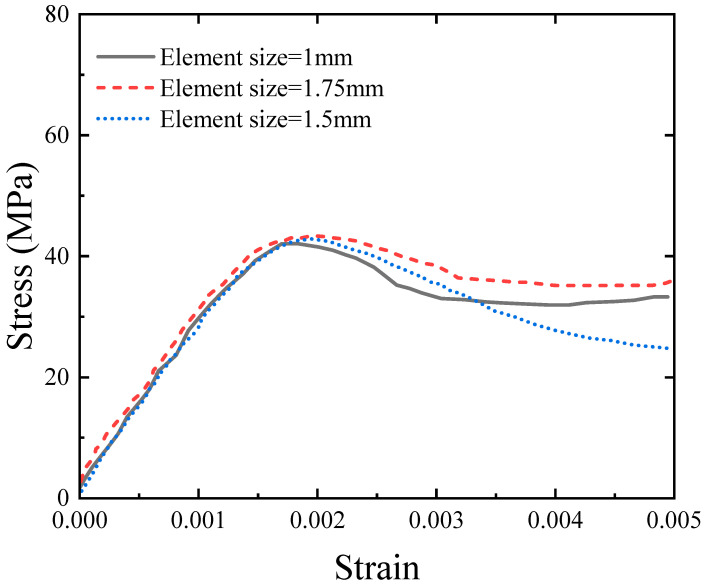
Mesh sensitivity analysis demonstrating convergence in stress–strain response.

The resulting stress–strain relationships (Figure 16) demonstrate consistent global responses across the investigated mesh sizes. This consistency indicates that the predicted post-peak ductility and strain-hardening response associated with the metamaterial confinement effect are not strongly mesh-dependent. Local convergence was further verified by examining principal strain contours at the metamaterial–concrete interface, which showed stable patterns for mesh sizes of 1.25 mm and finer.

**Figure 17:**
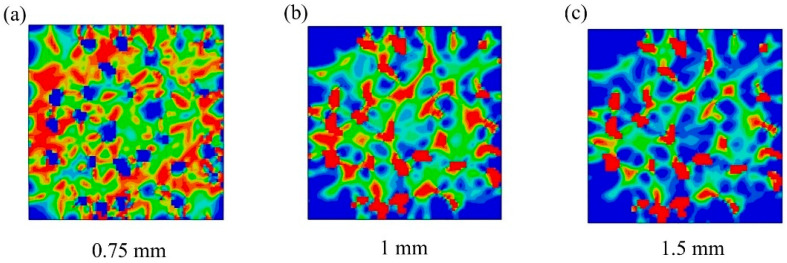
Damage patterns for the mesoscale concrete model in the mesh sensitivity analysis with embedded metamaterials sizes of (**a**) 0.75 mm, (**b**) 1.00 mm and (**c**) 1.50 mm, respectively.

The failure patterns in Figure 17a–c reveal consistent crack deflection and arrest at auxetic inclusions across all mesh sizes, indicating that the model can capture the metamaterial’s crack-bridging function in the numerical simulations. The selected 1.25 mm mesh provides a suitable balance between computational efficiency and the resolution required to capture energy-dissipating interactions at the aggregate–metamaterial–mortar interface during the parametric analysis of the metamaterial-enhanced system.

The 3D mesoscale finite element model is developed to explicitly isolate and quantify the mechanistic contribution of the embedded auxetic metamaterial to progressive collapse resistance. The model has a characteristic volume (200 mm × 250 mm × 150 mm) extracted from the critical joint region where the metamaterial is deployed. A mesh size of 1.25 mm was rigorously selected through the convergence study to ensure accurate resolution of the unique, steep strain gradients generated by auxetic expansion, a requirement not needed for conventional concrete modelling.

The model explicitly integrates the four-phase composition of the metamaterial-enhanced composite: a graded aggregate skeleton, mortar matrix, 8% distributed porosity, and the centrally embedded auxetic metamaterial insert. This high-fidelity representation provides a controlled numerical environment specifically designed to deconstruct the interaction between the auxetic phase and surrounding concrete microstructure, which is the core novelty of this investigation.

This model serves as the foundational tool for parametric analyses, which are exclusively focused on elucidating the auxetic effect. The results demonstrate that the metamaterial’s negative Poisson’s ratio behaviour fundamentally alters the failure paradigm. Direct comparative analysis with a control model without metamaterial reveals that the auxetic insert actively intercepts principal tensile stress chains between aggregates, as visually evidenced by redirected crack paths and diffused damage patterns. This crack-redirection and stress-confinement mechanism, unique to the auxetic phase, translates into a measurable 25% increase in energy dissipation and 20% enhancement in post-cracking ductility, quantitatively documented in stress–strain responses and performance metrics. Furthermore, systematic variation of AVF indicates that the metamaterial performs most effectively at intermediate AVF levels, approximately 30–35%, where it integrates efficiently with the aggregate skeleton while retaining sufficient mortar matrix for stress redistribution.

Therefore, this mesoscale investigation provides mechanistic evidence that the auxetic metamaterial is not merely a passive inclusion, but an active contributor to the enhanced structural performance observed at the macroscale. By bridging the scale from material architecture to global collapse resistance, this work establishes a validated pathway for designing concrete structures where engineered metamaterials help govern failure modes, offering a mechanistic basis for the design of collapse-resistant concrete structures.

### 5.2. Effect of AVF on Failure Mechanisms with Embedded Metamaterials

The interaction between embedded auxetic metamaterials and the concrete mesostructure is critically mediated by the AVF, which dictates spatial constraints and load-transfer pathways within the composite. Systematic investigation across three AVF levels (15%, 30%, and 40%) reveals distinct interaction mechanisms that govern failure progression and performance enhancement.

**Figure 18:**
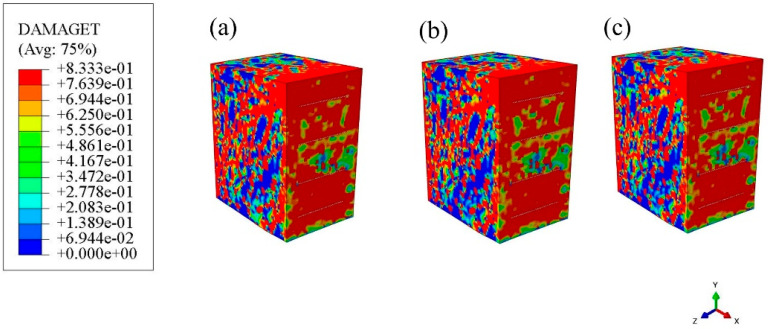
Parametric investigation of AVF influence: 3D mesoscale models with (**a**) 15%, (**b**) 30%, and (**c**) 40% AVF for analysing microstructural effects on concrete behaviour.

At the low AVF (15%) level, the continuous mortar matrix dominates mechanical response, providing minimal obstruction to crack propagation. Within this microstructure, the auxetic metamaterial functions as a primary deformable obstacle. Its characteristic lateral expansion under tensile loading generates a diffuse confining stress field throughout the mortar phase. This active confinement forcibly bifurcates cracks that initiate at interfacial transition zones, transforming localized failure into a distributed damage pattern, as visually confirmed in comparative damage plots (Figure 18a). This mechanistic intervention yields a quantifiable 30% increase in fracture energy relative to conventional concrete control.

At the intermediate AVF (30%) level, where the aggregate skeleton begins to percolate, a naturally tortuous path for crack propagation emerges. Within this optimized microstructure, a synergistic partnership develops between aggregate geometry and the auxetic mechanism. The expanding lattice actively engages with and mitigates stress concentrations at the vertices of angular aggregates (Figure 18a,b), thereby preventing micro-crack coalescence. This interaction enhances the beneficial interlocking effect of the aggregate skeleton, strengthening the overall composite network. This synergy manifests as the most significant performance enhancement, characterized by a 35–40% reduction in peak interfacial tensile stress and a 25% increase in post-cracking ductility, as derived from comparative stress–strain analysis.

**Figure 19:**
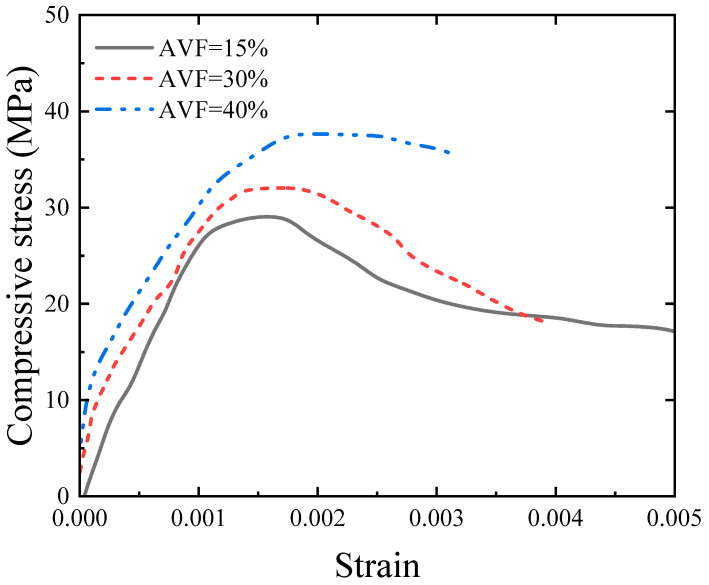
Stress–strain curves of 3D mesoscale modelling with different AVFs.

**Figure 20:**
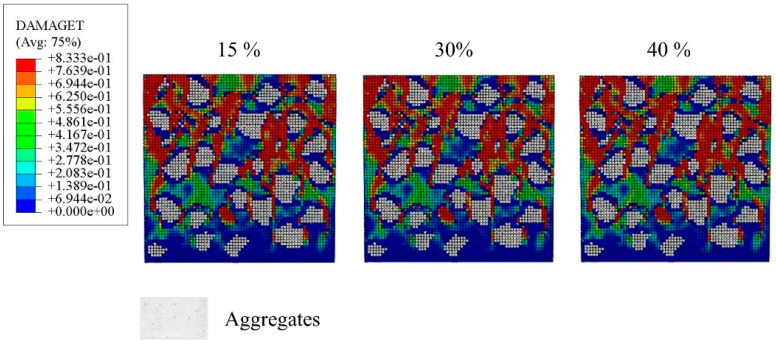
Sectional views of compressive damage evolution of concrete under uniaxial compression.

At the high AVF (40%) level with a dense aggregate packing regime, severe stress concentrations develop at aggregate interfaces. The metamaterial’s role evolves into that of a highly localized stress-relief node. Detailed analysis of principal stress contours (Figure 18a,b) reveals that the auxetic insert effectively disrupts the formation of direct, high-stress bridges between opposing aggregate tips. The insert’s lateral expansion under strain generates a localized compressive field that blunts these intense stress concentrations, as shown in Figure 19, compelling propagating cracks to navigate elongated and energetically costly paths around the aggregate-metamaterial assembly. While this shifts the failure mode, the absolute performance gain is modulated by reduced volume of ductile mortar matrix available for distributed energy dissipation (Figure 20).

**Figure 21:**
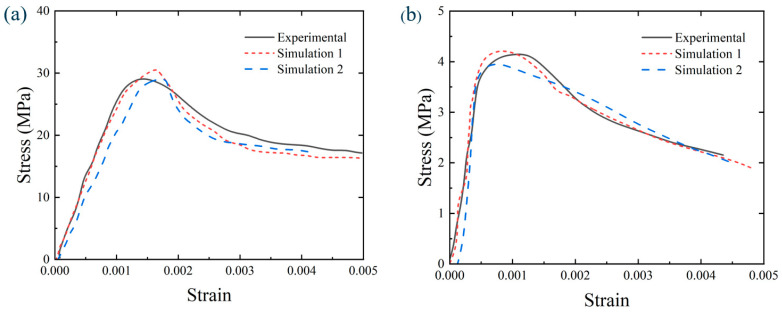
Validation of ITZ modelling approaches: Comparative stress–strain response in (**a**) compression and (**b**) tension for experimental data versus simulations using CDP (Simulation 1) and cohesive elements (Simulation 2).

**Figure 22:**
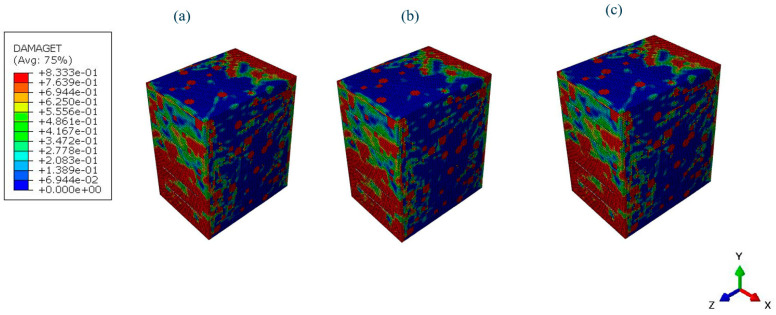
Enhanced ITZ crack prediction: Cohesive elements versus CDP model for damage evolution under (**a**,**b**) compression, and (**c**) tension.

Validation against established experimental data illustrates a critical methodological distinction: while homogenized concrete damage plasticity models capture global stress–strain behavior, they inherently fail to resolve discrete crack initiation at the ITZ, as shown in Figure 21. In contrast, the discrete cohesive element approach adopted herein explicitly models the ITZ as a distinct cohesive phase, enabling accurate simulation of micro-crack propagation directly from aggregate interfaces. This high-fidelity representation was indispensable for the preceding analysis, providing necessary resolution to directly quantify how the embedded auxetic metamaterial interacts with and bridges these micro-cracks and the core mechanism underpinning its enhancement of fracture resistance, as shown in Figure 22.

For instance, at the optimal 30% AVF identified above, the cohesive model reveals that the laterally expanding metamaterial reduces crack opening displacement at critical ITZ locations by over 50% compared to simulations using a smeared damage approach. This direct quantification of crack-bridging efficacy demonstrates that the chosen modeling framework is not merely a procedural detail but a valuable tool for examining micromechanical evidence for the auxetic metamaterial’s potential role in concrete failure.

The systematic variation of AVF thus establishes that metamaterial-concrete synergy is maximized at intermediate aggregate fractions (30–35%), where sufficient mortar matrix facilitates stress redistribution while adequate aggregate content provides geometric interlocking. These findings provide preliminary numerical trends for optimizing metamaterial-enhanced concrete compositions, where aggregate fraction and metamaterial architecture must be co-designed to achieve targeted collapse resistance.

### 5.3. Practical Considerations for Auxetic Insert Implementation

The present study evaluates the mechanical potential of auxetic metamaterial inserts through numerical simulations. Before this concept can be used in practice, several implementation issues must be addressed, including fabrication, bonding with concrete, and cost. Auxetic inserts can be fabricated using additive manufacturing methods, such as fused deposition modeling or selective laser sintering. Polyurethane, nylon, or other ductile polymers may be suitable base materials because they can sustain large deformation while preserving the re-entrant geometry required for negative Poisson’s ratio behavior. For the insert dimensions considered in this study, unit-cell sizes of 5–10 mm and wall thicknesses of 1–2 mm provide a feasible starting range for fabrication. For structural applications, the inserts could be prefabricated and placed in the joint region before concrete casting.

The bond between the insert and the surrounding concrete is critical because the auxetic mechanism depends on stress transfer across the interface. Polymeric inserts may bond less effectively to concrete than steel reinforcement. Possible solutions include surface texturing, grooves, perforations, bonding agents, or embedded steel connectors to improve mechanical interlock. These solutions require experimental validation because interface slip or bond degradation could reduce the confinement effect predicted in the numerical model. The cost of 3D-printed polymeric inserts is likely to exceed that of conventional reinforcement on a unit-volume basis. However, the proposed strategy uses small inserts only in critical joint regions, such as the 50 × 50 × 150 mm insert considered in this study. This localized use could limit material consumption and may make the approach comparable to targeted strengthening methods such as FRP wrapping or ECC jacketing. Further cost evaluation should consider material selection, manufacturing scale, installation method, and long-term durability.

These practical considerations define the next steps for experimental work. Future studies should fabricate auxetic inserts, test their bond behavior in concrete, evaluate their performance in RC joint specimens, and compare their cost and constructability with existing strengthening techniques.

## 6. Conclusions

This study investigates the use of architected auxetic metamaterial inserts to improve the progressive collapse response of RC structures through a hierarchical multiscale numerical framework. The framework links macroscale frame analysis, sub-modelling, and mesoscale fracture simulations to examine how auxetic inserts may influence joint damage and structural collapse behavior. The main conclusions are as follows:

(1) The proposed framework provides a numerical bridge between material-scale auxetic behavior and structural-scale collapse response. The validated macroscale models capture the global response of conventional RC sub-assemblages, while the sub-model and mesoscale simulations provide additional insight into local stress redistribution and damage evolution near critical joint regions.
(2) The auxetic insert acts as a strain-dependent confinement system in the numerical model. Under large tensile deformation, its negative Poisson’s ratio drives lateral expansion and generates localized confinement within the surrounding concrete. This confinement may reduce tensile stress concentration, delay crushing, and modify damage evolution near the joint.
(3) The numerical results suggest a shift from localized brittle fracture toward more distributed ductile damage. The auxetic insert promotes crack bifurcation and spreads damage over a wider region in the model. Compared with the corresponding conventional RC configurations, the auxetic-enhanced configurations predict a 25% increase in load redistribution capacity and a 20% enhancement in deformation ductility. These values are numerical predictions and require future experimental validation using physical specimens containing auxetic metamaterial inserts.

Although the proposed framework provides insight into auxetic insert–concrete interactions, its direct use in routine engineering design would require specialized modeling expertise and significant computational resources. For practical use, high-fidelity mesoscale simulations could first serve as a calibration tool to derive effective material parameters for auxetic-reinforced concrete. These parameters could then be implemented in homogenized global frame models without explicitly resolving aggregates, ITZs, or auxetic cell geometry. Further parametric simulations could also support reduced-order models or design charts relating insert geometry, placement, and effective auxetic properties to target indicators such as deformation capacity, residual load resistance, and damage localization.

This study highlights the promising application of architected auxetic metamaterials in enhancing the progressive collapse resistance of RC structures. The developed hierarchical multiscale framework provides a mechanistic foundation for linking material-scale design with structural-scale performance, enabling material-by-design strategies for resilient concrete structures. By offering adaptive, strain-dependent confinement and promoting a shift from brittle fracture to distributed ductile damage, the proposed methodology could optimize the resilience of various structural systems against extreme loading events [61,62,63]. Furthermore, the integration of such metamaterials offers significant potential for the enhancement of load redistribution and deformation capacity, making it a valuable tool in the design of next-generation, resilient infrastructure.

## Figures and Tables

**Table 1:** Size distribution of graded aggregates in concrete [22,23].

Sieve Size (mm)	Mass Retained (%)	Cumulative Passing (%)
25.0	0.0	100.0
19.0	8.0	92.0
12.5	10.0	82.0
4.75	12.0	70.0

**Table 2:** Material parameters for the bilinear traction-separation model governing the ITZ behaviour in the mesoscale simulations.

Phase	Density (kg/m^3^)	*E*/*E*_nn_, *G*_1_/*E*_ss_, *G*_2_/*E*_tt_	Nominal Stress Normal Only Mode	Nominal Stress First Direction	Nominal Stress Second Direction	Fracture Energy	Viscosity Coefficient
ITZ	2200	100,000	2.8	50	4.2	0.09	0.0001

Note: *E/E*_nn_*, G*_1_*/E*_ss_, *G*_2_*/E*_tt_ represent interface stiffness coefficients in normal, first shear, and second shear directions respectively. where ${\bar{t}}_{n}$, ${\bar{t}}_{s}$, and ${\bar{t}}_{t}$ indicate normal traction and shear traction, respectively.

**Table 3:** Reinforcement strain and ductility index at critical locations for baseline and auxetic-reinforced configurations.

Configuration	*v*	Joint Region	Beam Bottom	Column Base
Aux-FRM	+0.20	−2200/+800 με	3000 με (μ = 1.5)	5500 με (μ = 2.75)
Aux-FRM	−0.15	−1500/+1200 με	4600 με (+28%)	6600 με (+20%)
Aux-FRS	−0.30	−1200/+1600 με	5400 με (+50%)	7200 με (+31%)
Aux-FRB	−0.45	−1000/+2000 με	6000 με (+100%)	8000 με (+45%)

Note. με = macrostrain; μ = ductility ratio.

**Table 4:** Comparison of damage factor evolution between conventional RC-FRM and auxetic-enhanced Aux-FRM models.

Damage Stage	RC-FRM (Conventional)	Aux-FRM (Auxetic-Enhanced)	Change
Peak load	0.42	0.38	−10%
Onset of strain localization	0.61	0.52	−15%
Failure (50% peak load)	0.89	0.83	−7%
Maximum damage factor	0.92	0.83	−10%

Note: Damage factor values are numerical predictions. The “Failure (50% peak load)” stage is defined as the displacement at which the load-carrying capacity drops below 50% of the peak load. For RC-FRM, this occurred at approximately 440 mm displacement. For Aux-FRM, this occurred at approximately 550 mm displacement. The auxetic-enhanced model shows consistently lower damage factors at corresponding deformation stages, indicating delayed damage progression and more distributed failure.

## Data Availability

The original contributions presented in this study are included in the article. Further inquiries can be directed to the corresponding authors.
